# Automated Cobb Angle Measurements for Scoliosis Diagnosis and Assessment: AI Applications and Accuracy Enhancement Through Image Processing Techniques

**DOI:** 10.7759/cureus.66736

**Published:** 2024-08-12

**Authors:** Aurelio Pio Russo, Ylenia Pastorello, Lóránd Dénes

**Affiliations:** 1 Faculty of Medicine, George Emil Palade University of Medicine, Pharmacy, Science and Technology of Târgu Mureș, Târgu Mureș, ROU; 2 Department of Anatomy and Embryology, George Emil Palade University of Medicine, Pharmacy, Science and Technology of Târgu Mureș, Târgu Mureș, ROU

**Keywords:** cobb angle calculator, artificial intelligence, cobb angle, spinal deformities, scoliosis

## Abstract

Introduction

Scoliosis is characterized by an abnormal curvature of the spine in the coronal plane. Idiopathic scoliosis is the most prevalent type, though specific causes are sometimes identifiable. Genetic factors significantly influence adolescent idiopathic scoliosis (AIS), which is diagnosed through clinical and radiographic evaluations, primarily using the Cobb angle to measure curvature severity. The classification of scoliosis severity ranges from mild scoliosis, where sometimes the absence of pain is encountered, to moderate and severe, which is usually associated with lancinating pain. Early onset and high progression rates in idiopathic scoliosis are indicative of poorer prognoses.

Methods

The study analyzed 197 radiographic images from a private clinic database between December 2023 and April 2024. Inclusion criteria focused on anteroposterior images of the thorax and abdomen, excluding unclear and non-spinal images. Manual Cobb angle measurements were performed using RadiAnt DICOM Viewer 2020.2, followed by automated measurements using the Cobb Angle Calculator software. Discrepancies led to further image processing with enhanced color contrast for improved visualization. Data were analyzed using GraphPad InStat to assess error margins between manual and automated measurements.

Results

Initial results indicated discrepancies between manual and automated Cobb angle measurements. Enhanced image processing improved accuracy, demonstrating the efficacy of both manual and automated techniques in evaluating spinal deformities. Statistical analysis revealed significant error margins, prompting a refined approach for minimizing measurement errors.

Discussion

The study highlights the importance of accurate Cobb angle measurement in diagnosing and classifying scoliosis. Manual measurements, while reliable, are time-consuming and prone to human error. Automated methods, particularly those enhanced by machine learning algorithms, offer promising accuracy and efficiency. The integration of image processing techniques further enhances the reliability of scoliosis evaluation.

Conclusion

Accurate assessment of scoliosis through Cobb angle measurement is crucial for effective diagnosis and treatment planning. The study demonstrates that combining manual techniques with advanced automated methods and image processing significantly improves measurement accuracy. Such an approach is intended to support better clinical outcomes. Future research should focus on refining these technologies for broader clinical applications.

## Introduction

Scoliosis is defined as a curvature of the spine in the frontal or coronal plane. While scoliosis of unknown cause (idiopathic) is the most common classification, a specific cause can be identified in 20-25% of scoliotic patients [1]. The most common identifiable causes of scoliosis are related to injuries or infections of the spine, birth defects, and certain neuromuscular conditions, such as muscular dystrophy [2]. The overall prevalence of scoliosis in children and adolescents is in the range of 0.47-5.2%, being more common in females (female-to-male ratio of 3:1) and aggravating according to age. Adolescent idiopathic scoliosis (AIS) is mainly influenced by genetic factors, which play a significant role in both the occurrence and advancement of scoliosis. In the case of AIS, 97% of patients present familial connections, indicating a strong genetic component. In addition, individuals with Prader-Willi syndrome exhibit scoliosis in approximately 40% of the cases, further emphasizing the influence of genetic factors on the development of a pathological spinal curvature in specific populations [3].

The severity of the spinal curvature can be determined through the measurement of the Cobb angle. This is defined as the angle between two perpendicular lines traced at the upper margin of the uppermost involved vertebra and at the lower margin of the lowest involved vertebra. In order to confirm the diagnosis of scoliosis, the Cobb angle should be higher than 10° [4]. A Cobb angle falling in the range of 10°-25° indicates the presence of mild scoliosis. In case the angle ranges from 25° to 45°, the scoliosis is considered moderate. Severe scoliosis is diagnosed whenever the calculation of the Cobb angle provides a result greater than 45° [5]. Patients with scoliosis undergo classification based on the age of onset, severity, etiology, and curve type. Each of these categories exhibits distinct characteristics, including the rate of curve progression and the degree-pattern association of the deformity. In idiopathic scoliosis, factors such as a high rate of curve progression and early onset are used as negative predictive parameters, indicating an unfavorable outcome.

The female-to-male ratio has significant variations based on the Cobb angle: taking into consideration a Cobb angle value in the range of 11°-20°, the ratio is approximately 1.4:1, while a range of 21°-40° presents an increased ratio of 2.8-5.4:1. Comparing the severity of scoliosis and taking in consideration the female-to-male ratio, there is an extremely significant increase between the 1.4:1 ratio related to a Cobb angle of 11°-20° and a Cobb angle that is >40°, reaching a ratio of 7.2:1 [6-9]. Thoracic curves represent the most prevalent type, accounting for 48% of the cases, closely followed by lumbar curves at 40%. Less common are double curves, constituting 9% of the cases, and double thoracic curves, which are even rarer (3%). In total, approximately 80% of children diagnosed with scoliosis exhibit either thoracic or lumbar curves, with a prevalence of thoracic or lumbar curves in males and thoracic or double curves in females [7].

Scoliosis is broadly categorized into two main groups: idiopathic scoliosis and non-idiopathic scoliosis, the idiopathic one being diagnosed by exclusion of non-idiopathic causes [2,5,10]. Congenital scoliosis results from vertebral malformations such as hemivertebra, block vertebrae, or unilateral bony bar [6]. Neuromuscular scoliosis arises from an insufficient active stabilization of the spine, often seen in conditions like cerebral palsy, spinal muscular atrophy, or spinal cord injuries [11]. Mesenchymal scoliosis is due to a deficiency in the passive stabilizers of the spine. This is commonly associated with conditions like Marfan’s syndrome, osteogenesis imperfecta, and certain inflammatory diseases. In addition, it may manifest after thoracic surgical procedures, particularly those involving open-heart surgery [10]. Infantile scoliosis, juvenile scoliosis, adolescent scoliosis, and adult scoliosis are all based on the scoliotic onset age: infantile (0-3 years old), juvenile (4-10 years old), adolescent (11-18 years old), and adult scoliosis having a prevalence in adults over 25 years old, often caused by degenerative changes in the aging spine [3,10,11].

The diagnosis of scoliosis is usually based on signs and symptoms, being supported by clinical and paraclinical examinations. The signs of scoliosis are generally divided into two categories: structural signs and functional signs. The structural signs are all the signs limited to what the examiner can see, namely, unequal shoulder heights, shoulder blade prominence, waist asymmetry, and visible spinal deformities. Regarding the functional signs, those include muscular divergencies, range of motion (ROM) disturbances, gait and posture alterations, and pain resulting from the muscular divergencies, disc compressions, or even vertebral malalignment, and being directly related to the degree of spinal deformation [12].

Symptoms can usually result even from a slight deformity, ranging from a simple acute transitory pain to chronic persistent lancinating pain affecting the lower back and upper back and ultimately with a possibility of leading to respiratory symptoms. Not only the physical symptoms are to be taken into consideration, because many patients, especially adolescents, are very prone to social and psychological stress: seeing themselves in front of a mirror and looking at the spinal curvature, has enormous impacts on self-esteem, and there are chances of this resulting in anxiety and depression [5,10,12].

Regarding the clinical and paraclinical diagnostic methods for scoliosis, it is mandatory to mention a few important steps that lead to an accurate diagnosis. Physical examination, for example, is the most complete step if associated with imaging techniques. Of importance is Adam’s forward bend test, which is based on the patient bending forward, thus allowing the examiner to visualize the spine [13]. A careful medical history should be also assessed, to exclude any familial or past disease that could be associated with the development of spinal curvature; thus, if any disease results or any of the first-degree relatives present scoliosis or a spinal abnormality, genetic counseling is recommended. Studies proved that scoliosis is inherited in 30-80% of the cases [14,15]. Particularly, in adolescents with scoliosis, attention should be given to developmental patterns: a sudden increase in the patient’s height could lead to significant changes in the spinal curvatures and thus regular weight and height evaluations should be carried out [15]. Regarding the paraclinical examinations in the process of diagnosing scoliosis, these are commonly and routinely used to assess the spinal curvature and specifically the severity of a spinal deformity, meaning that by performing X-ray imaging, the examiner can have a clear image of the full spine. Therefore, via the Cobb angle calculation, scoliosis can fall in one out of 4 categories describing its severity. X-ray studies also enable the assessment of a potential vertebral rotation, information that is of extreme importance for the examiner to assess how the body is responding to a treatment [16].

This study aims to assess the clinical applicability of artificial intelligence (AI) for automated Cobb angle measurement and scoliosis assessment. Evaluation of existing AI algorithms and optimal alternatives to avoid human implications in the measurement methods is pursued.

## Materials and methods

Study design

A total of 197 patients were included in this study. Their radiographic images were obtained from SC Dora Medicals SRL, a private clinic in Târgu Mureș, Romania, over the period of December 2023-April 2024 and subsequently analyzed. The only inclusion criteria that were followed regarded the areas of interest displayed in the radiographic images: anteroposterior images involving the thorax and the abdomen were included. Unclear images and images of specific anatomical regions (e.g., shoulder, knee, hand, and foot) were excluded. These criteria were followed in order to have an optimal spinal visualization.

All of the images underwent manual Cobb angle measurement using the software RadiAnt DICOM Viewer 2020.2 (Medixant, Poland). In order to reduce the measurement error, the images were examined three times. After the manual calculations for the Cobb angle, all the values were transferred in Microsoft Excel (365 v. 2402, Microsoft Corporation, United States) and divided into two categories: manual thoracic and manual lumbar. Subsequently, automated Cobb angle measurements were performed by processing all 197 images via an open-source Software, namely, “Cobb Angle Calculator,” designed specifically for automated Cobb angle measurement. Once the images were processed, the Cobb angle values were obtained. By analyzing the preliminary results, since a discrepancy in the two sets of data (manual and automated) was noticed, a further step was introduced. This step was fundamental for a correct diagnosis, and it was based on processing the raw X-ray images by enhancing the contrast to have a better visualization of the bony structures and thus the vertebral column. Once all the images were processed and contrast was enhanced to an optimal degree, they were exported and examined via the Cobb Angle Calculator again, hence ultimately having three sets of data. This approach aimed to explore the accuracy and reliability of both manual and automated techniques in evaluating spinal deformities and their severity, contributing to a better understanding of their applicability in daily clinical practice.

Statistical analysis was carried out using the software GraphPad InStat3.0 (Dotmatics, United Kingdom) to assess the error margins between the manual measurements and the automated ones. A comprehensive evaluation of the data and procedures was undertaken to identify strategies for minimizing the margin of error to the greatest extent possible.

Cobb Angle Calculator

The open-source software Cobb Angle Calculator (v1) is a Windows program developed using the You Only Look Once (YOLO) architecture, a sub-convolutional neural network (CNN) category based on object identification. The software is coded in Python 3.8, a programming language that allows the use of machine learning, neural networks, and deep neural networks to focus on the ability of these branches of AI to train themselves to achieve better results over time. Different extra functions are coded in the software, namely, the polynomial curve fitting (PCF), and the Hough transform (HT).

Machine Learning

Machine learning is a branch of AI based on algorithm development, instructing software to learn from data and to make various predictions without the need to be programmed for it [17]. Therefore, in our case, giving a sample image, the software could try to predict each time where the next vertebral body would be situated, and it kept trying the prediction until it reached an accuracy of >90%. Once this threshold was reached, the next vertebral body was identified. Deep learning is a branch of machine learning that focuses on neural networks by building a number of imaginary layers in order to learn complex pathways. Its functioning follows the complexity of the human brain, by building blocks of information and interconnecting them to find the safest and quickest way to reach a set goal [17,18].

Neural and Deep Neural Networks

A neural network, as mentioned, follows the human brain functioning. It consists of blocks or nodes that are interconnected and organized into layers. Let’s imagine that we are using a neural network, and we want to train our studied model to detect the vertebral bodies: the first step would be to give input to one of the imaginary neurons. After defining the format input and the target, this neuron divides the received input into three different blocks: training data, test data, and validation data. Successively and at the same time, these three blocks of information are sent to three different neurons, each one with the instructions to create a forward feed. These three neurons then proceed to make predictions for the vertebral body position, and at the same time, they send a forward feed; each of the three neurons toward three more neurons; hence, in the second training round, a number of nine neurons will be involved. The procedure repeats over and over again until one of the neurons is giving a feed with zero errors. That path is then taken into consideration and the network starts to train itself.

CNN

A standard neural network cannot be used in our study. Even though vertebral identification looks promising, standard neural networks can only be used for blocks of information and data. For our model that is based on analyzing X-ray images, a CNN is needed, which is a type of deep neural network used to process visual information [19]. A CNN is more complex than a standard neural network, and by breaking it down, different levels involving different functions would result. The convolutional layers are the ones, which are filtering the input data and are basically the ones that directly visualize the image and detect all the needed information. All the gained information that are processed in the convolutional layers are then transmitted to the pooling layers, which are working on the 2D or 3D dimensions of the information in order to reduce to the minimum terms the complexity of the information. Since a neural network needs to be imagined as a properly functioning human brain, we must assume that there is no existence of information linearity, meaning that the CNN needs somehow to gain consciousness of the tridimensionality of the information. This is achieved by the activation functions, which introduce the CNN to the non-linearity concept [17-19]. After these layers are repeated a couple of times, the gained information has to be transmitted among the neurons. For this purpose, the fully connected layers (FCLs) have been developed, in which all the neurons from one layer are connected to each neuron in the next layer.

YOLO

YOLO is a CNN architecture for object detection first introduced in 2016. YOLO can identify and locate objects within an image by using single-shot detection. It splits the image and builds predictive boxes for object location in real time. YOLOv3, the used CNN in our studied method, includes 53 convolutional layers, hence the ability to be fast and efficient [20]. Since two of the most common problems encountered in vertebral mapping are the end vertebral plate and the vertebral twisting identification, the pyramid feature of YOLO is of extreme importance. This feature allows the identification of objects with different resolutions and scales by dividing the image as if it were a pyramid: we can imagine the base of the pyramid as the most visible structure in a picture and the apex of the pyramid as the furthest points of a picture, with a lower resolution. It extracts different layers of information from the CNN (from the imaginary pyramid), thus detecting the objects regardless of the size, the distance, or the resolution.

PCF

The PCF is a mathematical formula used to generate a polynomial function that represents a set of points [21]. This can be used in the analysis of the spinal curvature or in the prediction of it. The process of building up a polynomial function representing a spinal curvature is based on various steps, the first one being based on some spinal measurements such as vertebral body position, vertebral edge position, spinal curvature at different levels, and the angles in between each vertebral body. Once all the data are gathered, the values are introduced in the polynomial function template, trying to get the minimum error possible between the actual spinal measurements and the predicted ones. The polynomial function is expressed as follows:

$f(x) = a_n x^n + a_{n-1}x^{n-1} + \cdots + a_2 x^2 + a_1 x + a_0$

where x is the independent variable; a_n,a_(n-1),…,a_1,a_0 are the coefficients of the polynomial; n is the degree of the whole polynomial function, representing the maximum x power in the equation [21].

HT

HT is an image-extraction technique used to detect target objects in an image and transform the detected points into coordinates. The HT works by dividing the objects from an image into spatial segments and by representing a number of points in these segments. Normally, tracing a line to delimit an object would be the easiest and fastest procedure, but since the vertebral bodies and edges have a tridimensional and irregular form, by analyzing them in spatial segments, better accuracy is achieved. The HT, in this case, is harmoniously working with YOLO, thus avoiding the common problem encountered with HT alone, which is the difficult identification of the vertebral endplate. YOLO can receive as information all the points traced by HT and then compare them with the ones measured during the pyramid process. Thus, instead of creating a classical 2D Hough space, a tridimensional aspect is analyzed.

General Functional Framework

**Figure 1:**
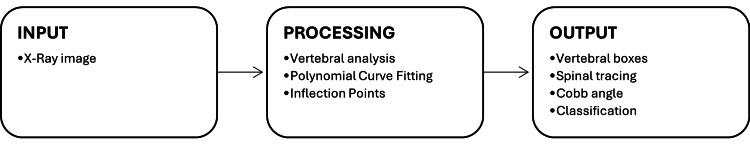
General diagram of the proposed method.

The diagram of the proposed method illustrates the three main involved steps in Figure 1. For the input section, an X-ray image is uploaded to the software. In the processing step, the software crops the image to closer visualize the vertebral bodies, and it analyzes them by retaining information regarding the body and the edge positions. After detecting this information and using the HT to translate the detected points into coordinates, the PCF is built, thus allowing the tracing of a line following the spinal midline. The inflection points detected are used to detect the first and last involved vertebrae. Regarding the output, by using spatial points and coordinates, the vertebral boxes are traced respecting the vertebral edges and variations. Right after this, the spinal midline that was calculated by using the PCF is drawn on the image. The inflection points that were used to detect the peripherally involved vertebrae are now used to trace the lines for the Cobb angle calculation, and based on this, the classification of scoliosis is given. The three possible options for the output classification are thoracic scoliosis, lumbar scoliosis, or a combination of the two.

Image Processing Framework

**Figure 2:**
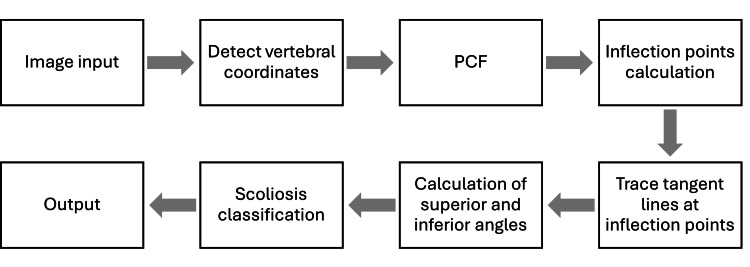
Diagram of the image processing steps. PCF: polynomial curve fitting

The image processing procedure consists of eight steps shown in Figure 2, from the input given by the user to the output given by the algorithm. The first step is to provide the software with an X-ray image, which is clear enough to visualize the vertebral column. During this step, the software (which is already trained at this point) tries to detect each vertebra and assign coordinates to it. Once the vertebrae are found, boxes are drawn around each vertebra, and the center of each box is calculated. Once the algorithm has gained information regarding the center of each vertebra, the PCF is able to build the function related to the spinal curvature, which is drawn on the image. Inflection points are successively calculated, showing the variations in the vertebral bodies, and by combining the PCF with the inflection points, tangent lines can be traced in order to calculate the Cobb angle. After the algorithm received the Cobb angle as feedback, the value is now undergoing scoliosis classification, thus entering a subroutine for the quality assessment.

Classification Subroutine

**Figure 3:**
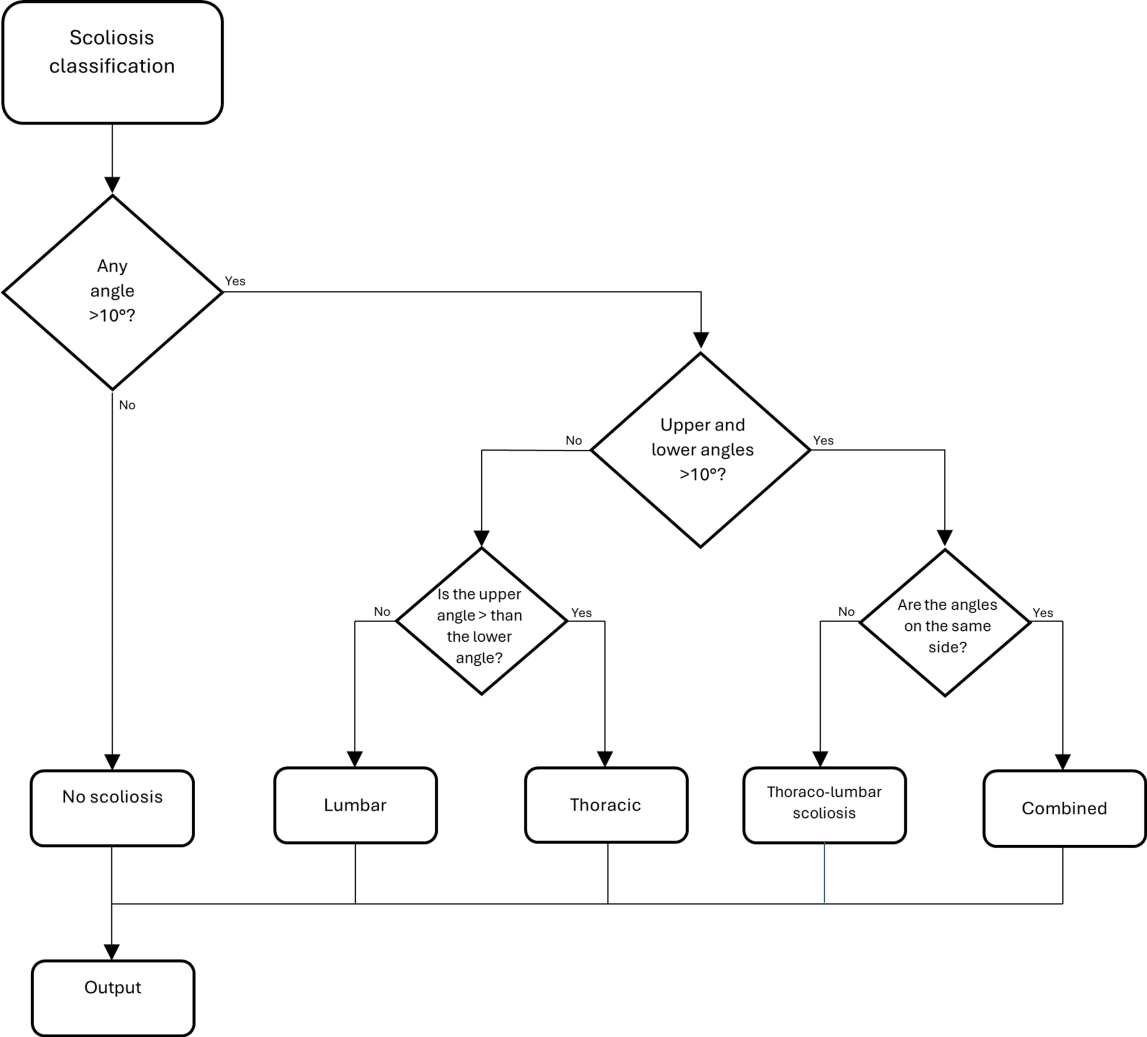
Diagram for scoliosis classification.

The subroutine for the classification of scoliosis is shown in Figure 3. When reaching the first decision box, the algorithm checks if there is the presence of any angle >10°, and if no angle above 10° is detected, the patient does not present scoliosis. Hence, the output box is reached, and the algorithm gives us negative feedback. In case any of the measured angles is greater than 10°, the second decision box will be activated, where it will be checked if both angles measure >10°. In case the condition is not met and only one angle is greater than 10°, we move on to the third decisional box, where it will be checked if the upper angle is greater than the lower angle or vice versa. In case the upper angle is greater than the lower one, the option of thoracic scoliosis is chosen, while if the upper angle is smaller than the lower one, lumbar scoliosis will be displayed. If the condition in the second decisional box is met and both angles are greater than 10°, the algorithm moves on to the fourth decisional box, where the question is whether the angles are toward the same side or not. In case the angles are shown toward different sides, we would have thoracolumbar scoliosis, whereas if the angles are toward the same side, we have combined scoliosis.

Training Framework

**Figure 4:**
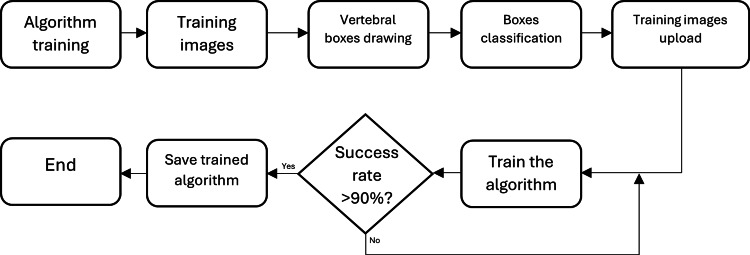
Algorithm training framework.

The algorithm was trained by using 194 radiographic images, which were obtained from a dataset published on SpineWeb, which is an online platform offering different training image datasets. The obtained images had 900 x 1900 pixels dimension, and thus, each examined X-ray after the training period was re-sized to fit the proportions. For each of the 194 training images, vertebral boxes had to be added manually, so that the algorithm would understand that each box corresponded to a vertebra (boxes classification). Right after the classification, all the images were sent as input to the algorithm and what YOLO was doing was to look for all the vertebral boxes placed on each image. Once all the data were recorded, the algorithm proceeded to take each image and divide it into many sectors in order to have higher accuracy in the prediction stage. The next step was then to try to predict all the areas in which the boxes could be situated and also to accurately predict all the boundaries of each box in a specific sector. This predictive process continued until the algorithm had a success rate higher than 90%. Once the threshold was reached, the trained algorithm was saved and exported into the image processing algorithm (Figure 4).

Software Results

**Figure 5:**
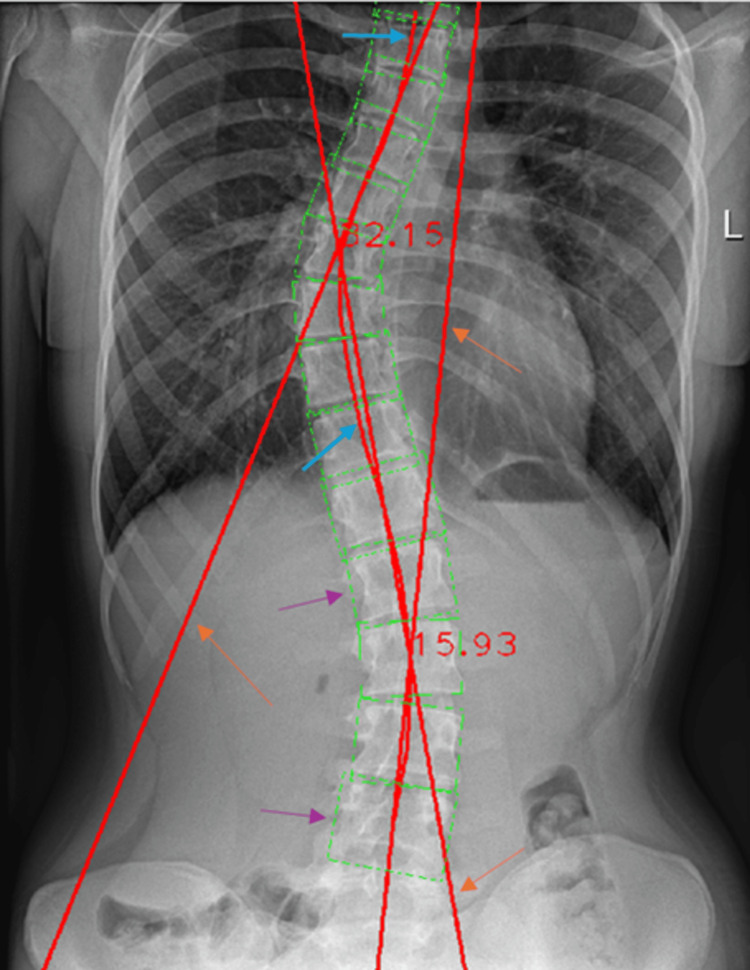
Resulting elaborated X-ray image. 1) Orange arrows showing the tangent lines from which the Cobb angle is calculated, 2) purple arrows showing the vertebral boxes, 3) light-blue arrows showing the central spinal line calculated via the polynomial curve fitting.

Once an X-ray image is uploaded into the software, all the algorithms are performed, and the final image is sent back to the user; the tangent lines for the Cobb angle calculation, the spinal curvature, and the vertebral boxes are shown. In Figure 5, all the mapping lines can be seen: all the red lines are used either to calculate the Cobb angle or to assess the center of the spine, while the green lines are used to map the anatomy of each vertebra by creating a full box following the margins of each the vertebra.

Ethical approval

Ethical approval no. 2680/15 dated December 2023 was obtained from the Research Ethics Committee of the University of Medicine, Pharmacy, Science and Technology “George Emil Palade” of Târgu Mureș.

## Results

Statistical results

**Figure 6:**
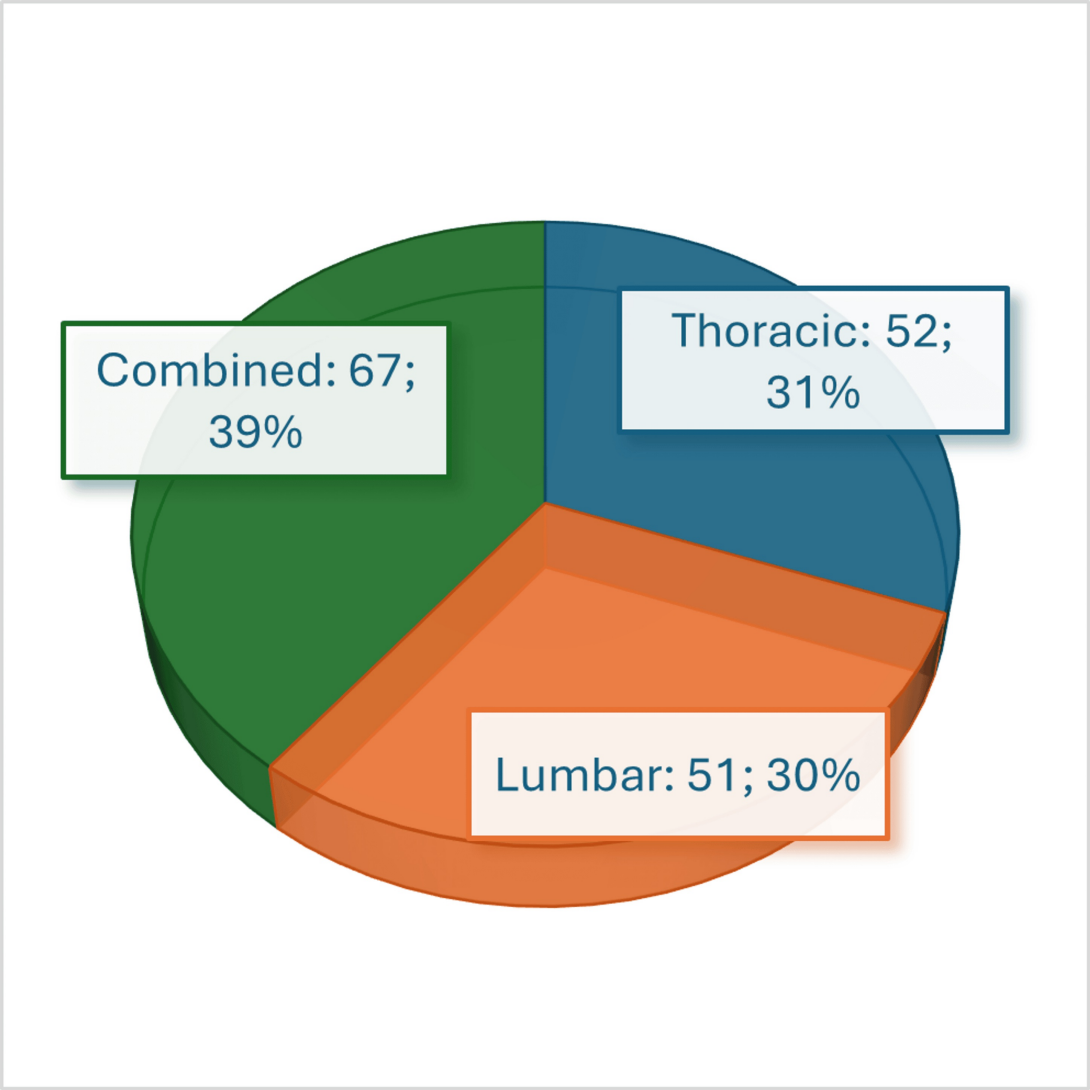
Patients' division according to the presenting spinal curvature(s).

The radiographic images of 197 patients were examined via the traditional Cobb angle measurement, leading to the diagnosis of scoliosis in 170 patients. Fifty-two patients presented a thoracic deformity, 51 patients had a lumbar deformity, and 67 patients presented combined thoracic and lumbar deformities (Figure 6).

**Figure 7:**
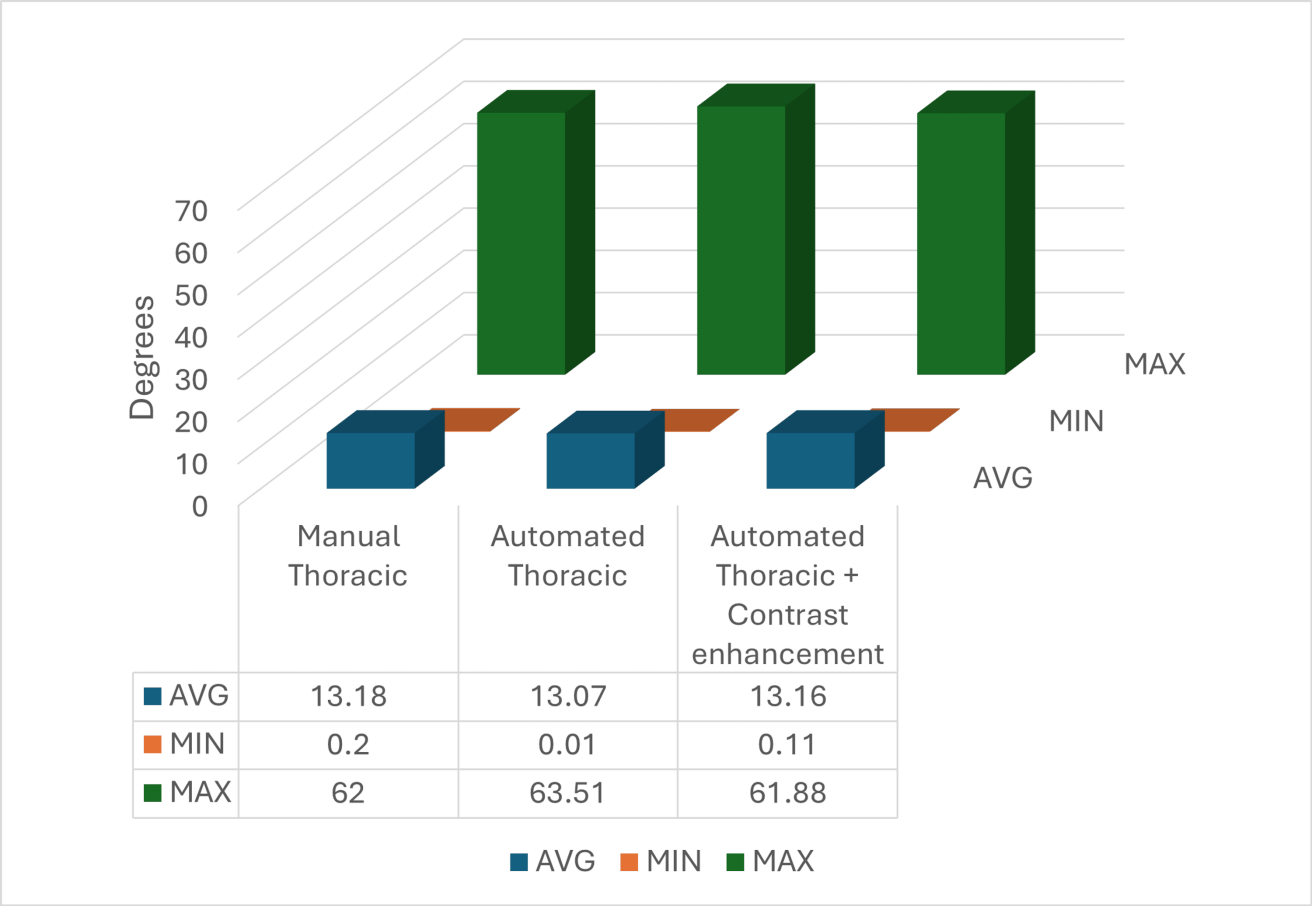
Average, minimum, and maximum values of the thoracic measurements pursued manually, automated, and with contrast enhancement. AVG: average, MIN: minimum, MAX: maximum

Using GraphPad InStat, data regarding the average, the minimum, and the maximum Cobb angle were obtained, based on the three sets of data. Figure 7 shows the thoracic measurements, including the manual thoracic measurements, automated thoracic measurements, and automated thoracic measurements with image contrast enhancement.

For the manual thoracic measurements, a Cobb angle average of 13.18° was obtained. A minimum value of 0.2° was obtained, and a maximum value of 62°, regarded as severe scoliosis was obtained. For the automated thoracic measurements, the average Cobb angle was 13.07°, the minimum 0.01°, and the maximum 63.51°, showing a difference in average compared to the manual measurements of 0.11°, a 0.19° difference between the minimum, and 1.51° for the maximum values. Ultimately, for the automated thoracic measurements with contrast enhancement, values of 13.16° for the average, 0.11° for the minimum, and 61.88° for the maximum were obtained. By comparing these values with the automated thoracic and the manual thoracic values, the differences were calculated. A difference in the average of 0.02°, 0.09° for the minimum, and 0.11° for the maximum was calculated between the manual thoracic measurements and the automated thoracic measurements with contrast enhancement, having thus closer values to the manual measurements compared to the automated ones without contrast enhancement. The values were closer as follows: 0.09° for the average, 0.1° for the minimum angle, and 1.39° for the maximum angle, showing higher accuracy and closer values to the manual ones.

A second procedure limited to the lumbar segment of the vertebral column was performed following the same steps as the previous one. The average Cobb angle and the minimum and maximum values were calculated and compared over each other, showing once again that the automated measurements with contrast enhancement were closer to the manually calculated ones when compared to the automated values without contrast enhancement.

**Figure 8:**
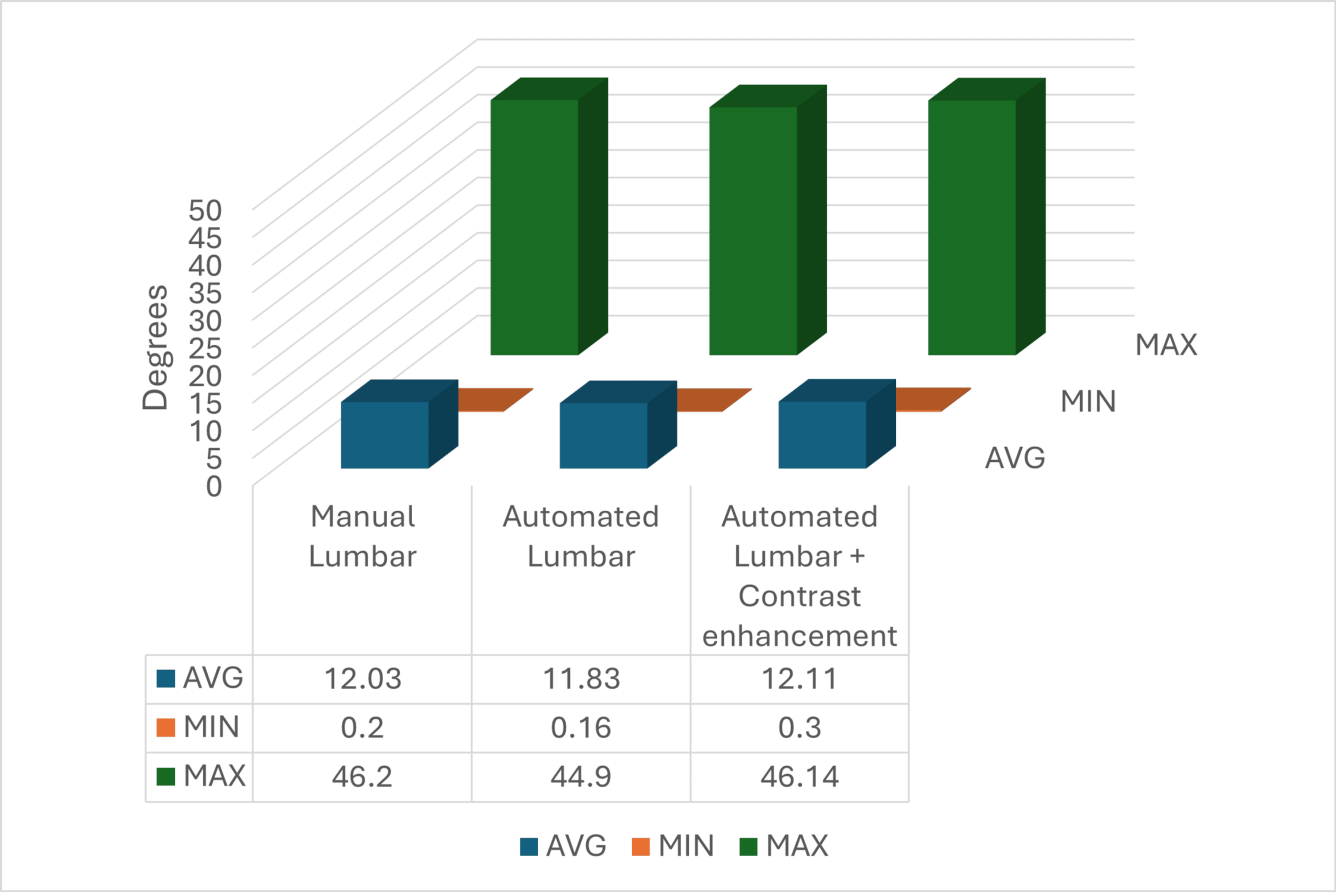
Average, minimum, and maximum values of the lumbar measurements pursued manually, automated, and with contrast enhancement. AVG: average, MIN: minimum, MAX: maximum

For the manual lumbar calculations, the average Cobb angle value was 12.03°, the minimum was 0.2°, and the maximum was 46.2°. Regarding the automated values without contrast enhancement, the average was 11.83°, the minimum was 0.16°, and the maximum was 44.9°, thus resulting in a difference compared to the manual values of 0.2° for the average, 0.04° for the minimum, and 1.3° for the maximum. For the automated lumbar measurements with contrast enhancement, the calculated values were 12.11° for the average, 0.3° for the minimum, and 46.14° for the maximum, leading, after calculating the differences by comparing them with the automated measurements without contrast enhancement, to closer values to the original manual lumbar measurements by the mean of 0.12° for the average and 1.24° for the maximum. Regarding the minimum measured value, no enhancement was shown. By contrast, a more distant value was measured, with a worsening of 0.06° (Figure 8).

Ultimately, after the roentgenograms were processed firstly with the RadiAnt software and then with the automated Cobb Angle Calculator, statistical analysis using “GraphPad InStat” was carried out demonstrating that the difference in the accuracy of the two methods is extremely significant (p = 0.0002), thus rejecting the null hypothesis. After the first stage of statistical analysis conduction, as shown in the figures above, each radiographic image underwent a contrast-enhancing process to improve the visualization of the bony structures, namely, the thoracic spine when measuring the thoracic curvature, the lumbar spine when measuring the lumbar curvature, and both combined when the aim was to simultaneously measure the thoracic and lumbar curvatures. After collecting all the data resulting from the automated software analyzing each contrast-enhanced image, the data was compared a second time. This time, the obtained p-value of 0.3019, considered not statistically significant, confirmed our null hypothesis.

Clinical results

Concerning the clinical practical applications for the automated Cobb angle measurement, three different sets of measurements were performed, with the first set involving radiographic images of thoracic scoliosis, the second one involving lumbar scoliosis, and the third one involving combined scoliosis (thoracic curvature plus lumbar curvature). Each image was processed a total of four times, including the contrast-enhancement process, starting with a manual measurement by using the software RadiAnt DICOM Viewer 2020.2. The second step was based on introducing the radiographic image into the software “Cobb Angle Calculator” for the automated measurement of the Cobb angle. The former original image was then re-processed on RadiAnt, undergoing a process of contrast enhancement in order to better visualize the spine at the thoracic segment. Ultimately, the fourth step consisted of processing the contrast-enhanced image by using the automated software. This whole process is valid for all three sets of images, with the only difference being in the contrast-enhancing step, which was based on which segment of the spine needed to be examined: for the first set of images, the thoracic spine needed to be examined, and thus the contrast-enhancing procedure was directed toward a thoracic segment spinal bony visualization; for the second set of images, the process was performed by enhancing the image toward a better visualization of the lumbar spinal segment; and for the third set of images, both thoracic and lumbar spinal segments were taken into account, so that the better bony visualization of both segments was aimed at.

**Figure 9:**
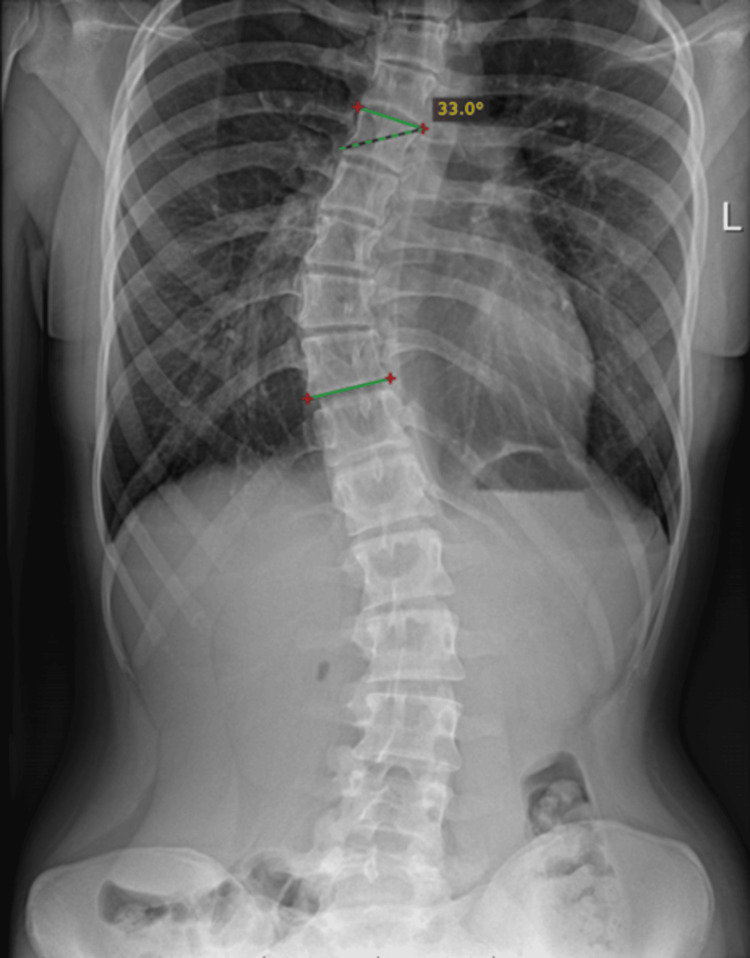
Manual Cobb angle measurement of the thoracic spinal curvature.

A simple raw radiographic image of the thorax and abdomen was examined, without contrast enhancement, and by using the Cobb angle gold standard measurement: the manual one. The Cobb angle was calculated three times, showing a value of 33° ± 0.04° (Figure 9).

**Figure 10:**
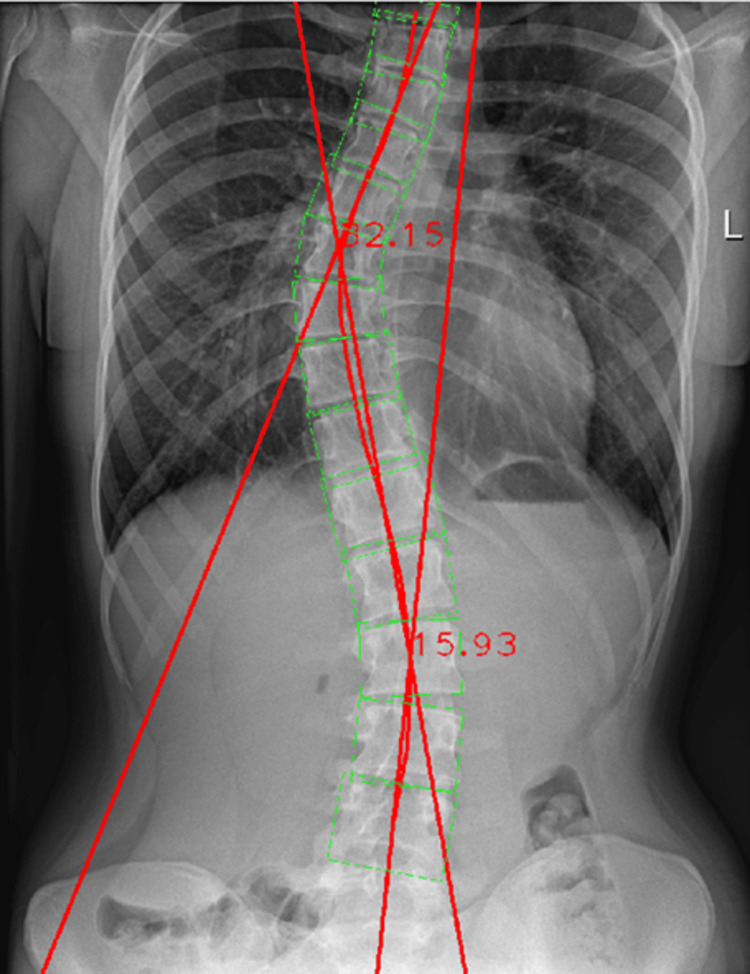
Automated Cobb angle measurement of the thoracic spinal curvature.

The same radiographic image was then processed by the Cobb Angle Calculator software. The resulting Cobb angle from the automated measurement was 32.15°, showing a difference of 0.85° when compared to the manual measurement (Figure 10).

**Figure 11:**
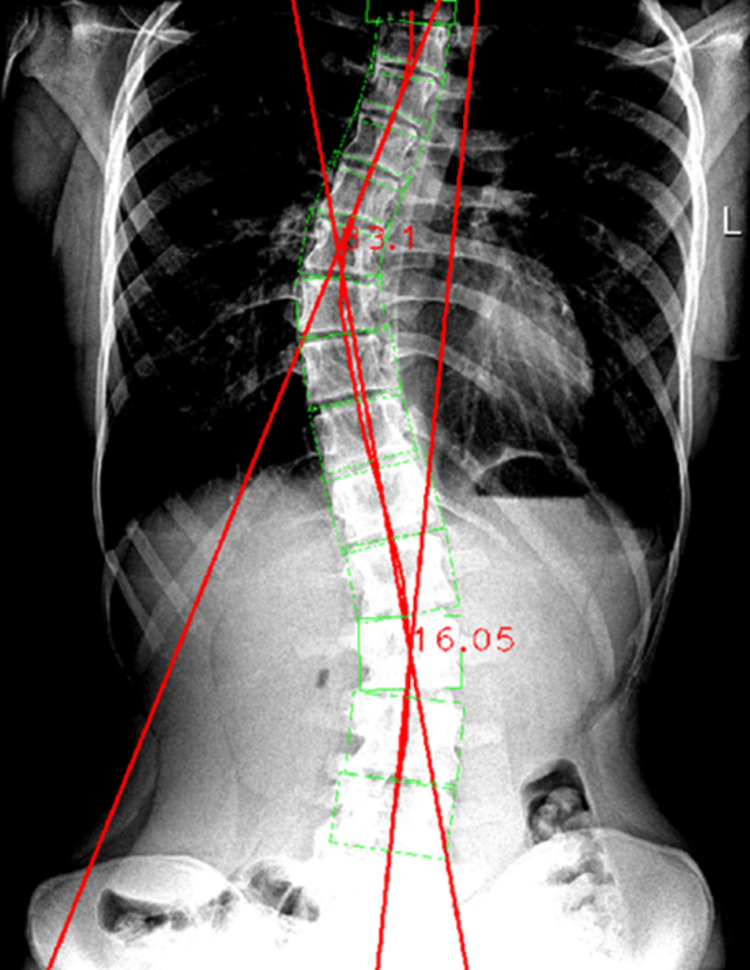
Automated Cobb angle measurement of the thoracic spinal curvature with contrast enhancement.

The raw original radiographic image was then re-introduced on the RadiAnt software in order to proceed with the contrast-enhancing procedure. Then, the image was again analyzed by the Cobb Angle Calculator software showing a Cobb angle thoracic value of 33.1°, thus only showing a 0.1° difference when compared to the manual measurement. The difference between the contrast-enhanced image and the non-enhanced one, when compared to the manual measurement, was 0.75°, thus showing a noticeable improvement (Figure 11).

**Figure 12:**
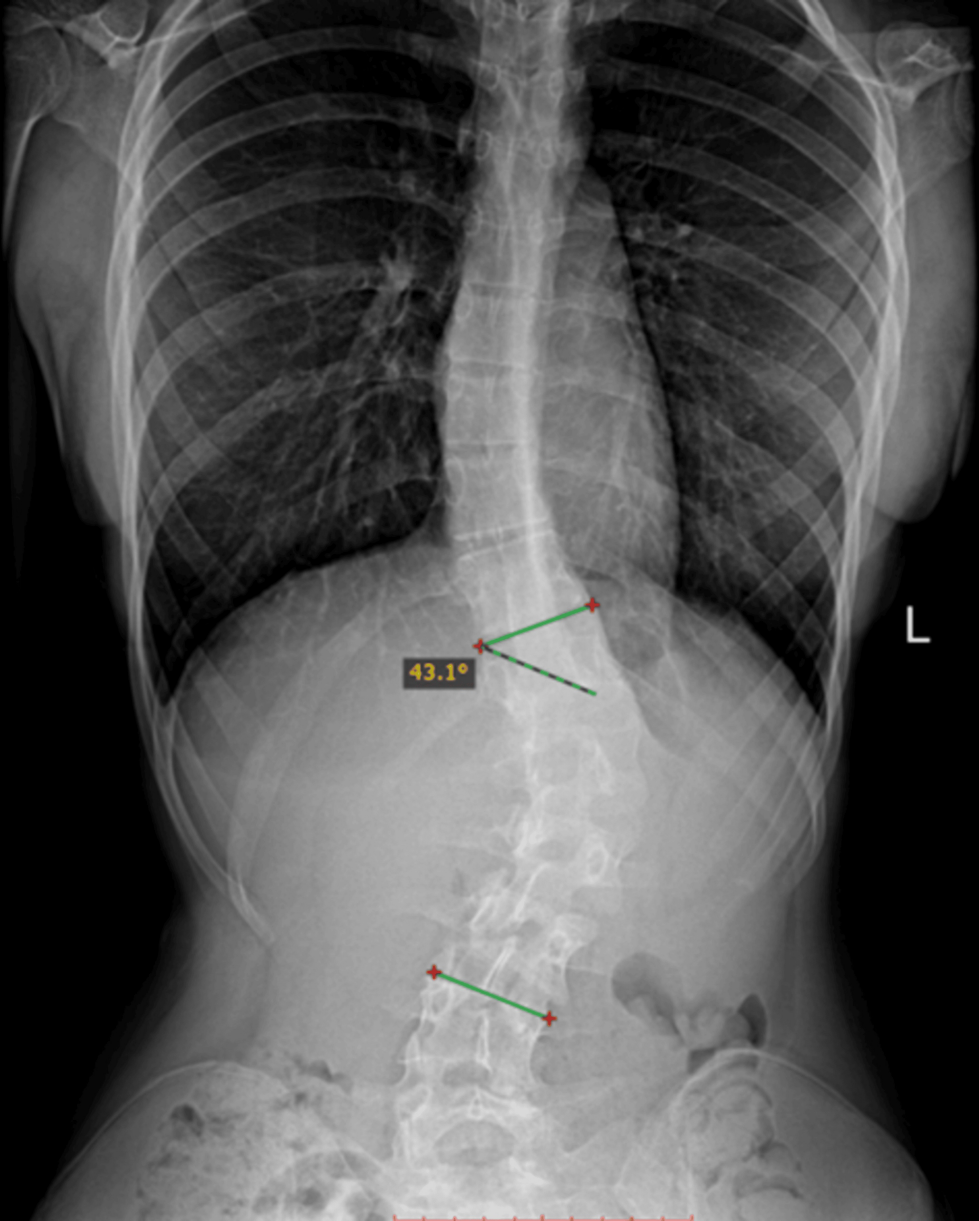
Manual Cobb angle measurement of the lumbar spinal curvature.

The exact same steps were successively followed for the lumbar spinal measurements. The raw image was manually assessed by using the RadiAnt software and demonstrating a lumbar Cobb angle of 43.1° ± 0.06° (Figure 12).

**Figure 13:**
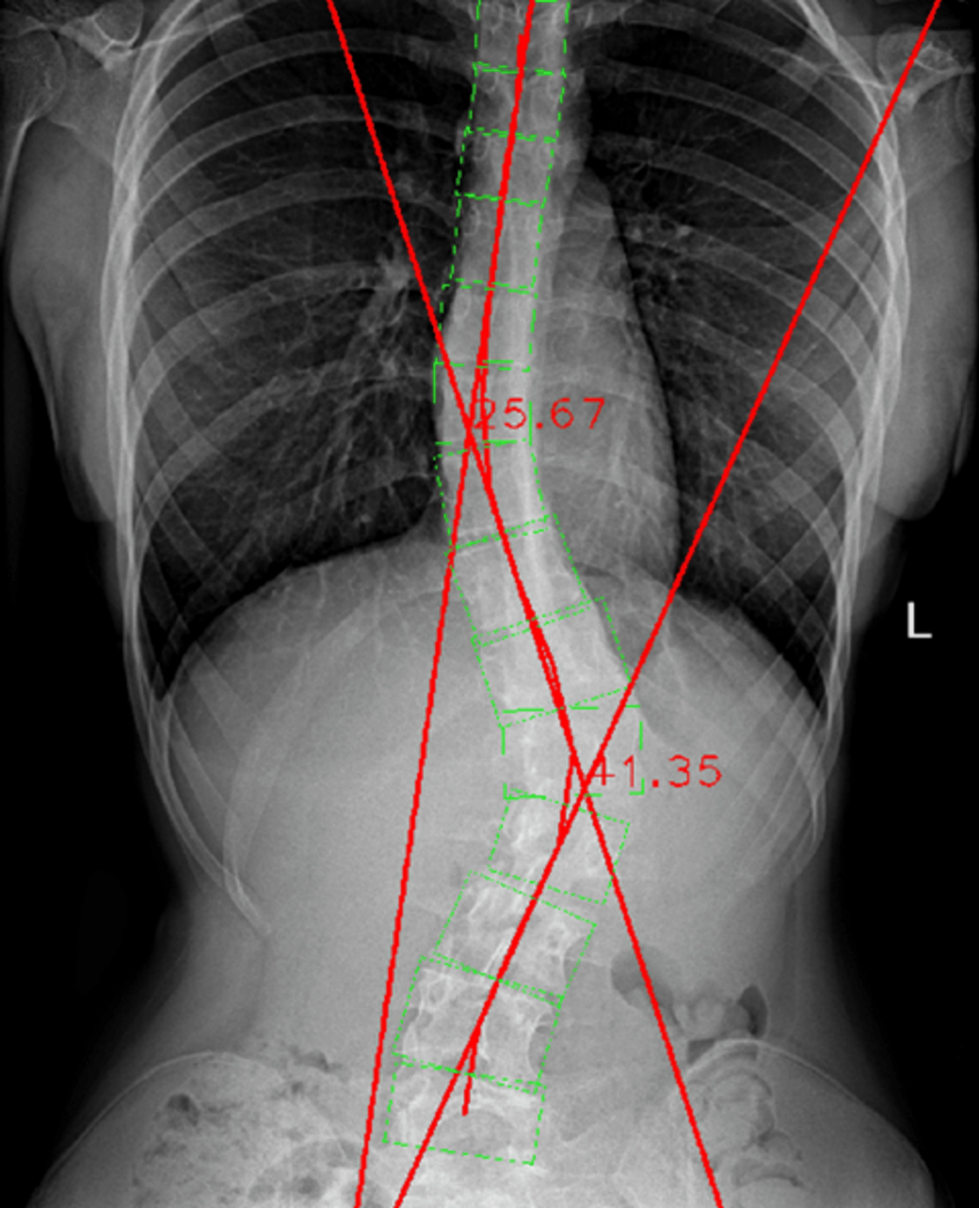
Automated Cobb angle measurement of the lumbar spinal curvature.

The radiographic image was then processed by the Cobb Angle Calculator software. The resulting angle was 41.35°, showing a difference of 1.75° when compared to the manually measured one (Figure 13).

**Figure 14:**
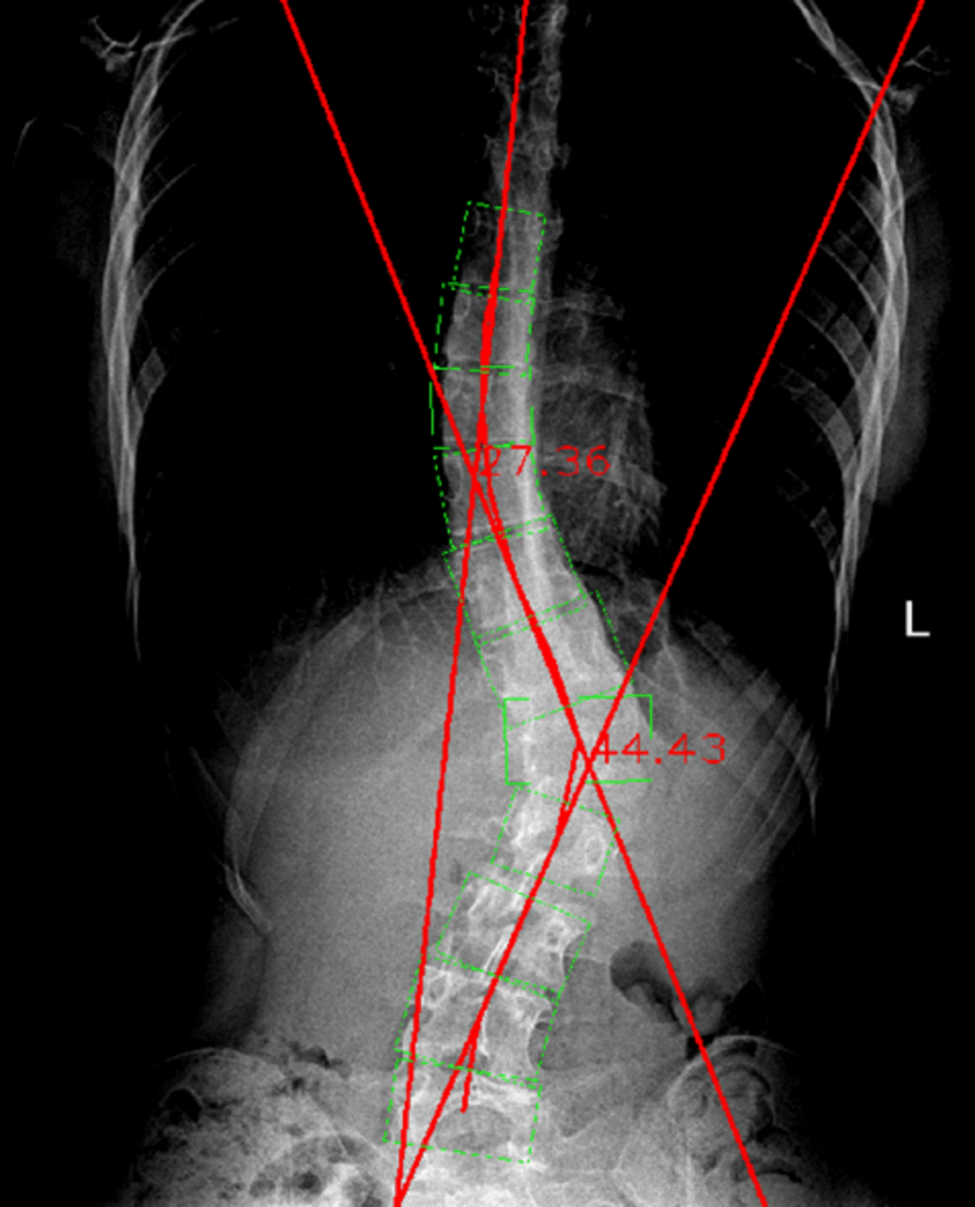
Automated Cobb angle measurement of the lumbar spinal curvature with contrast enhancement.

After the automated measurement, the raw image was processed a second time (or analyzed) by RadiAnt in order to perform the contrast-enhancing procedure. This time, the lumbar segment of the spine was prioritized, so the contrast was modified to an extent by which the lumbar vertebral bodies were more visible. Then, via the Cobb Angle Calculator, the resulting lumbar Cobb angle value was 44.43°, thus only showing a 1.33° difference, when compared to the manual measurement. By comparing the two automated measurements in relation to the manual one, the automated measurement with contrast enhancement resulted to be 0.42° closer to the manual measurement when compared to the automated one without contrast enhancement (Figure 14).

**Figure 15:**
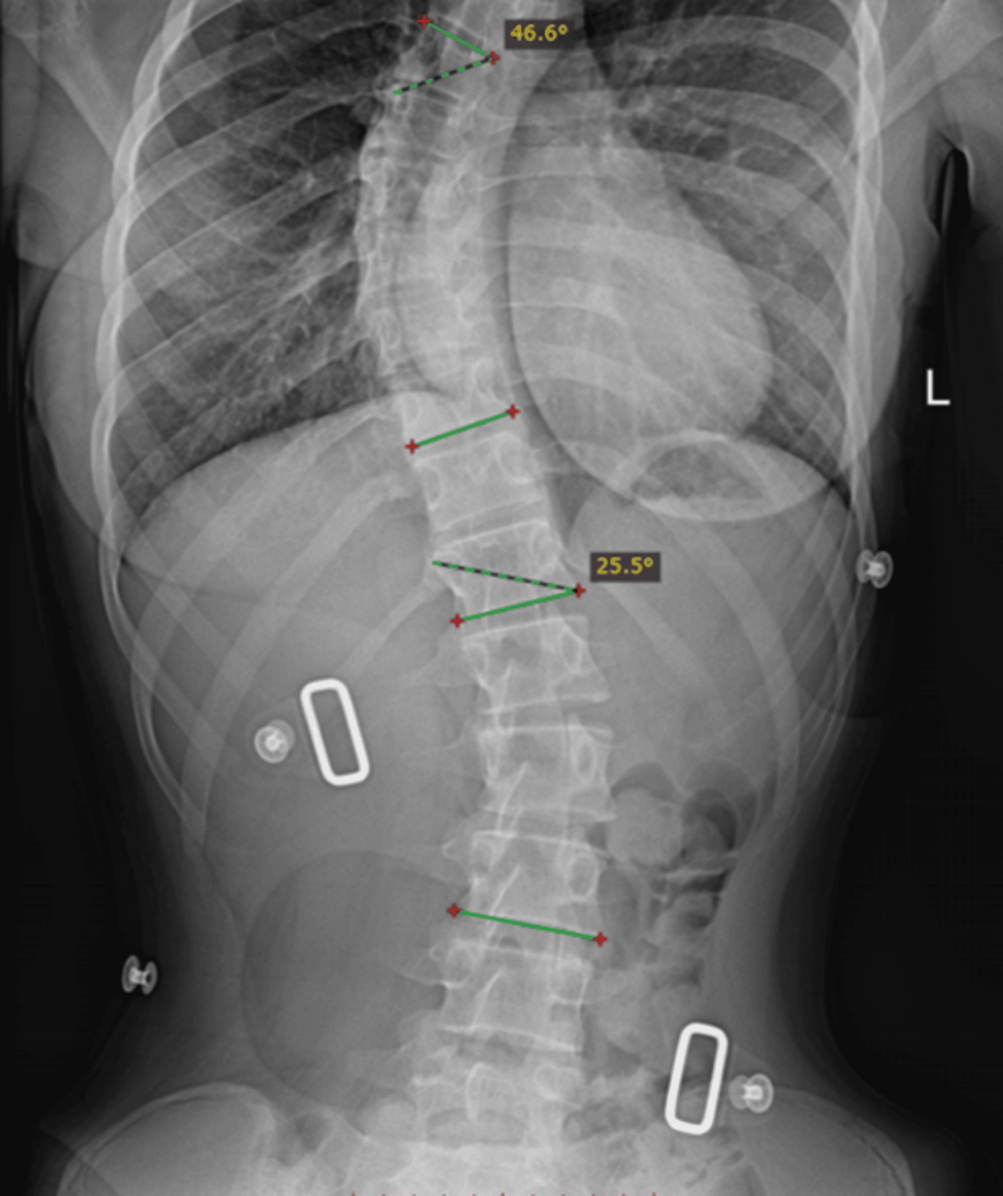
Manual Cobb angle measurement of the thoracic and lumbar spinal curvatures.

A third set was examined, this time without performing a thoracolumbar division. The raw image was manually analyzed with the RadiAnt software, and both the thoracic and lumbar curvatures were measured, leading to values of 46.6° and 25.5°, respectively (Figure 15).

After the manual calculations of both the thoracic and lumbar spinal curvatures, the image was processed via the Cobb Angle Calculator software, which provided the results for the curvatures. The thoracic curvature showed a value of 55.61°, being 9.01° away from the manual measurement.

**Figure 16:**
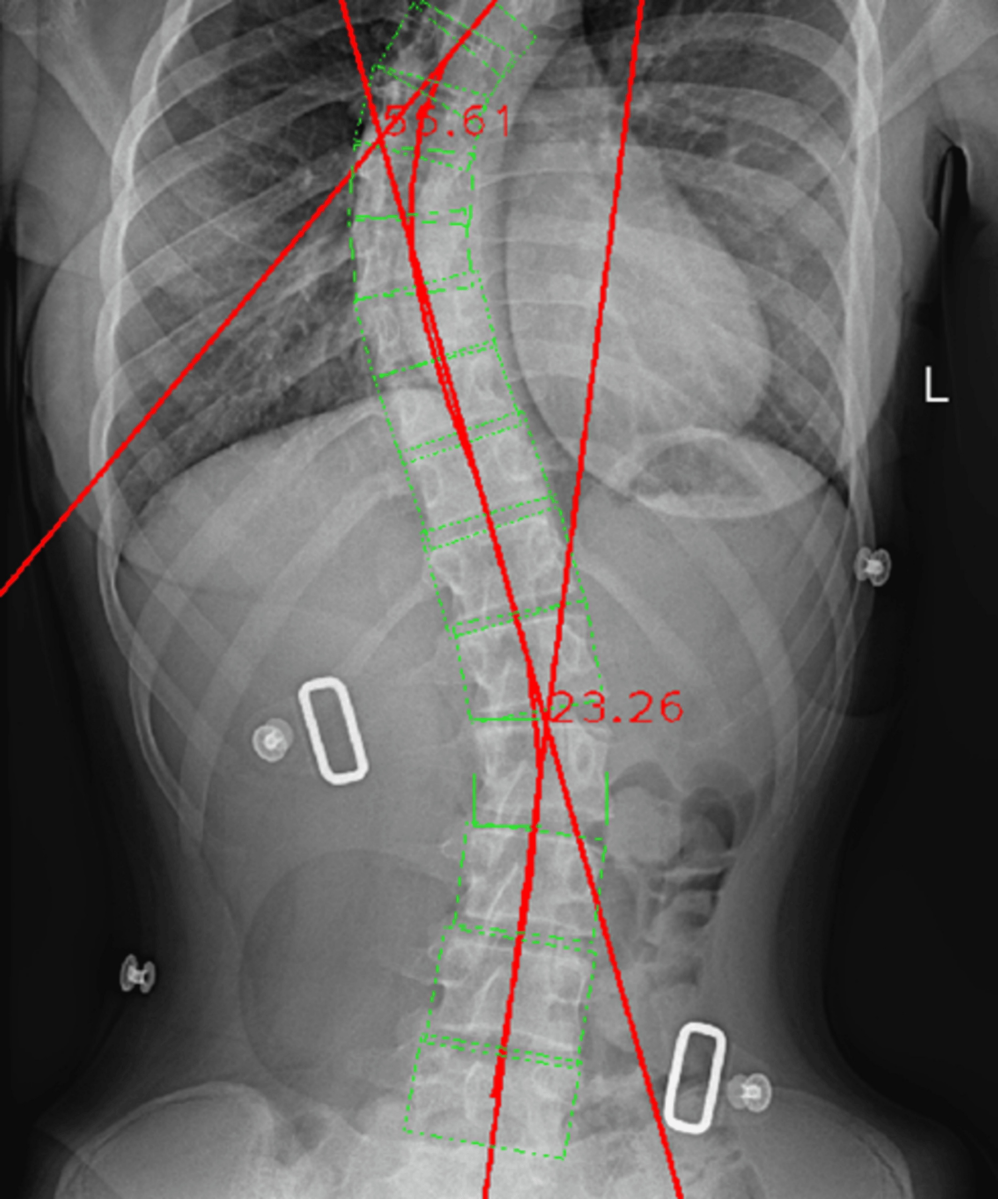
Automated Cobb angle measurement of the thoracic and lumbar spinal curvatures.

Regarding the lumbar spinal segment measurement, a value of 23.26° was shown, presenting a 2.24° difference when compared to the manual measurement (Figure 16).

Successively, the contrast-enhancing procedure was performed, trying to find the perfect balance between the thoracic and the lumbar segments. The vertebral body segmentation boxes were still overlapping, but in this case, the lateral protrusion of the boxes was not exaggerated. Thus, the resulting thoracic Cobb angle value was 48.79°, this time being only 2.19° away from the manual measurement. If we compare the two automated values, the thoracic automated Cobb angle value with contrast enhancement resulted to be 6.82° closer to the manual thoracic measured angle when compared to the automated thoracic one without contrast enhancement.

**Figure 17:**
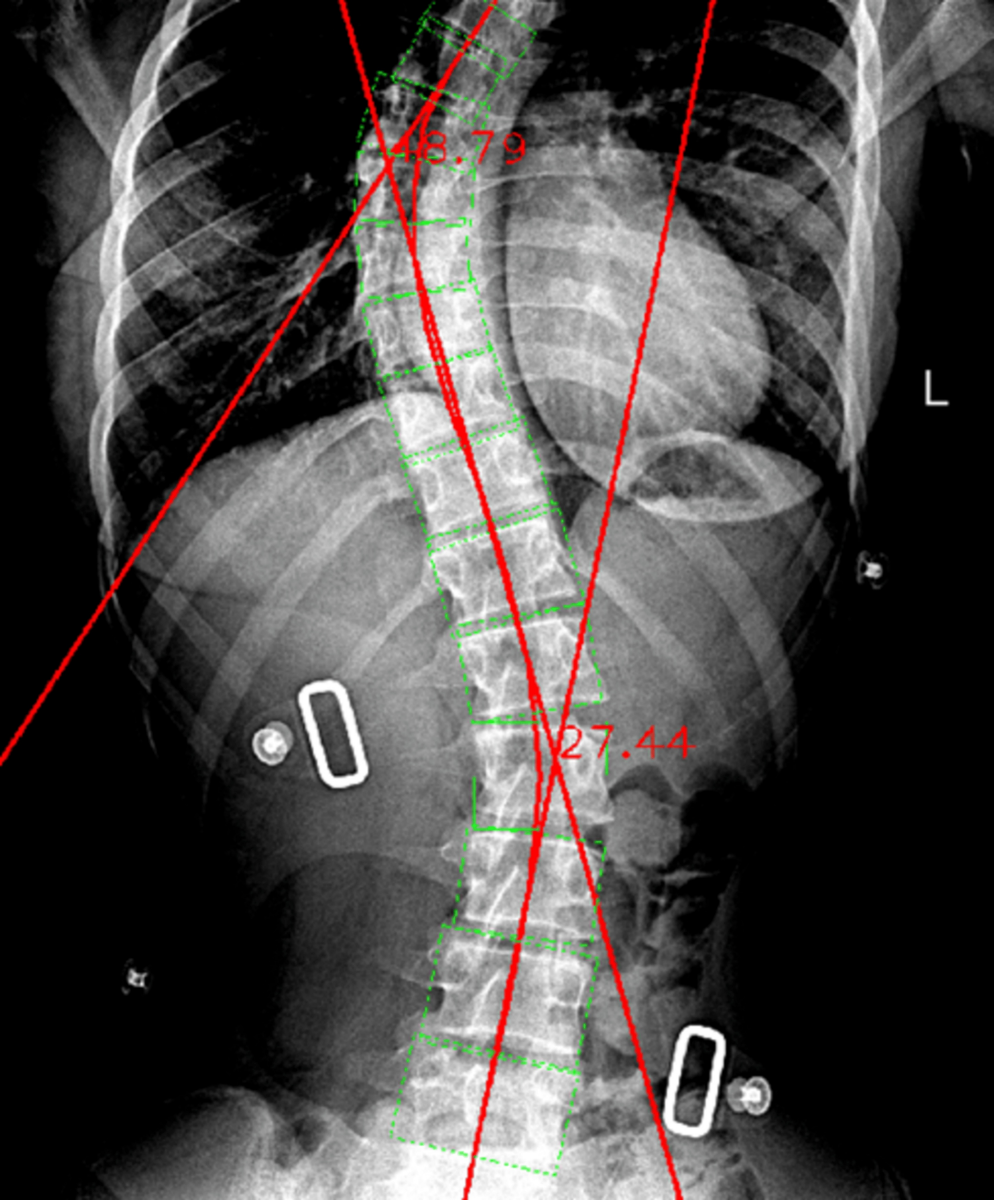
Automated Cobb angle measurement of the thoracic and lumbar spinal curvatures with contrast enhancement.

Regarding the lumbar measurement with contrast enhancement, the resulting value is 27.44°, showing a 1.94° difference when compared to the manually obtained value. When comparing the two automated values in relation to the manually measured ones, a 0.3° improvement is shown when considering the radiographic image with contrast enhancement (Figure 17).

## Discussion

Over the past 10 years, numerous different new approaches have been developed in order to accurately assess the degree of spinal deformation in patients with scoliosis. All these approaches were aiming to avoiding the time-consuming gold standard procedure, which is the manual assessment of the Cobb angle [22]. Even though all the current developing diagnosis procedures aim to maintain high accuracy and decrease the procedure time, X-rays remain time-consuming practices. Each new method involves X-ray automated assessment via several software, which approaches the radiographical image in a different way. Chockalingam et al. were among the first ones to develop their own software for Cobb angle measurement: In 2001, they created a software by using MATLAB software, which allowed them to select a section of the spine and make the required measurements [23]. The limit of the software consisted of the fact that the operator had to decide which section of the spine was undergoing the Cobb angle calculation, thus becoming a subjective procedure prone to errors. In the software used in this study, there is no possibility of any manual interaction from the operator; thus, once the radiographical image is uploaded, the software proceeds to identify the vertebral bodies and calculate the Cobb angle. In 2011, another kind of approach was explored by Anitha et al., where different steps were involved in order to accurately measure the Cobb angle and to reduce to the minimum extent the operator’s error [24]. Their new approach involved four steps from the image preprocessing to obtaining a clear image, to the HT [24,25]. Even if this procedure provided an extensive improvement, the main problem encountered was the identification of the vertebral endplates. Nevertheless, in their proposed method, the need of human intervention was needed, by manually selecting the first and last involved vertebras. This problem can be overcome by adding to the software a feature that can identify anatomical deformities of the vertebrae and tilting of vertebral bodies.

In order to automate the image-enhancing process, further improve the spinal identification, and gain more accuracy in the Cobb angle measurements, Wahyu et al. developed a pre-processing method for image enhancement and involved deep learning architecture for spinal segmentation by using a CNN [26]. Their method consisted of three stages, with each stage further divided into multiple steps, starting with Stage 1 including the detection of the features, their classification, and image cropping. In the second stage, segmentation of the spine, vertebral detection with their center and corners was performed, and in the last stage, the curvature of the spine was examined. Their study is extremely promising, with only three limitations being the need for a clear image, the fact that the software was able to measure only one single major spinal curvature, and the non-clinical setting in which the software was tested. These features exclude any possibility of double or multiple curvature measurements [26].

Our studied method, despite the need for a contrast-enhancing procedure that could be implemented, is already able to identify two major spinal curvatures. In case of multiple curvatures, a third measurement stage should be developed, which would measure any further curvature beyond the thoracic and lumbar ones. Even by integrating a neural network to allow the software to train itself to detect the vertebral bodies and spinal segmentation, the software was not developed in such a way to perform the image pre-processing with contrast enhancement by itself, hence still needing human intervention. The first step of statistical analysis compared the manual measurements to the automated ones leading to a p-value of 0.0002, considered extremely significant. Such a result points to the rejection of the null hypothesis, demonstrating that the automated calculated values are distant from the manual ones, meaning that the automated software displays low accuracy and reliability. Both sets showed net improvement when considering the contrast-enhancing procedure, but this process is obviously time-consuming and requires high accuracy when it comes to the bony structures’ visualization. A second step of statistical analysis was then carried out and the obtained p-value was 0.3019, which is considered not statistically significant, hence confirming the null hypothesis and proving the valuable aid of AI on Cobb angle quantification as an alternative to manual evaluation. Some measurements returned quite abnormal results, but it is to be mentioned that some patients presented vertebral body twisting, and the automated software, not able to distinguish different structures with similar color grading, overlapped the drawn boxes for the vertebral map. Even though a slight overlap of the vertebral body map does not extremely affect the Cobb angle measurement, in Figure 16, it is quite clear that the green boxes are laterally exceeding the vertebral bodies, thus resulting in abnormal feedback. This procedure can be elaborated by using deep machine learning and neural networks so that the software could find the perfect image contrast that would allow the best vertebral bodies visualization.

To the best of our knowledge, this is the first time a camera interface was implemented in the “Cobb Angle Calculator “software. Such a camera interface allows any type of camera, including X-ray machines, to be connected to a computer, thereby enabling a direct transfer of images to the software, saving time, and reducing the need for human interaction. However, issues with image contrast remain.

Limitations of the study

Future work should focus on the development of an automated contrast-enhancement technique capable of regularly preparing images for precise Cobb angle computation free from human supervision. In addition, advances in software competence should include improved algorithms for identifying spinal malformations. In particular, the ability to detect and measure various spinal curvatures and vertebral body twisting are two important elements in the evaluation of scoliosis.

## Conclusions

To date, there is no published, free, open-source software that allows operators to obtain spinal measurements without any manual intervention. Despite the numerous software published over the last four years, none of them is currently able to pre-process an image to reach an optimal spinal visualization. Our studied method is currently at the same stage, with the need for a preliminary image elaboration step, which can potentially be developed and thus could make the software completely automated.

Our method demonstrated extremely accurate results compared to the gold standard for scoliosis measurement, ensuring reliable spinal curvature assessments. Nonetheless, without contrast enhancement, measurements can vary, leading to potential misdiagnoses. Therefore, a pre-processing routine should be added to the software to improve overall reliability and prevent scoliosis misdiagnosis.
